# Developing an integrated framework of problem-based learning and coaching psychology for medical education: a participatory research

**DOI:** 10.1186/s12909-015-0516-x

**Published:** 2016-01-05

**Authors:** Qing Wang, Huiping Li, Weiguo Pang, Shuo Liang, Yiliang Su

**Affiliations:** School of Psychology and Cognitive Science, East China Normal University, Shanghai, China; Department of Respiratory Medicine, Shanghai Pulmonary Hospital, School of Medicine, Tongji University, Shanghai, China

## Abstract

**Background:**

Medical schools have been making efforts to develop their own problem-based learning (PBL) approaches based on their educational conditions, human resources and existing curriculum structures. This study aimed to explore a new framework by integrating the essential features of PBL and coaching psychology applicable to the undergraduate medical education context.

**Methods:**

A participatory research design was employed. Four educational psychology researchers, eight undergraduate medical school students and two accredited PBL tutors participated in a four-month research programme. Data were collected through participatory observation, focus groups, semi-structured interviews, workshop documents and feedback surveys and then subjected to thematic content analysis. The triangulation of sources and member checking were used to ensure the credibility and trustworthiness of the research process.

**Results:**

Five themes emerged from the analysis: current experience of PBL curriculum; the roles of and relationships between tutors and students; student group dynamics; development of self-directed learning; and coaching in PBL facilitation. On the basis of this empirical data, a systematic model of PBL and coaching psychology was developed.

**Conclusions:**

The findings highlighted that coaching psychology could be incorporated into the facilitation system in PBL. The integrated framework of PBL and coaching psychology in undergraduate medical education has the potential to promote the development of the learning goals of cultivating clinical reasoning ability, lifelong learning capacities and medical humanity. Challenges, benefits and future directions for implementing the framework are discussed in this paper.

## Background

Problem-based learning (PBL) is essentially a strategic learning system that represents a major shift in the educational paradigm from teacher-centred to student-centred learning. It aims to enhance collaborative, contextual, integrated, self-directed and reflective learning [1]. Although PBL may take various forms in different institutions, it is generally built on the following principles: relevant, authentic problems form the basis of teaching and learning; students as the central players and active seekers of knowledge; and learning through collaboration and discussions [2]. Essential features of PBL include an interdisciplinary approach, authentic activities that are valued in the real world, ill-structured problems, students’ collaboration, individually collected information, groups’ decision-making process, discussion of principles and goals, and self- and peer-assessment [3].

As an instructional method pervasively employed in medical education [4], PBL has been adopted in Mainland China since the mid-1980s as a means of cultivating students’ practical learning capacities and, ultimately, promoting lifelong learning [5–9]. Chinese medical schools have been making efforts to explore their own PBL approaches based on their educational conditions, human resources and existing curriculum structures [10–16]. Most Chinese medical schools select and incorporate some essential features of PBL into their existing curriculum as a hybrid model in which the majority of teaching is done through didactic lectures and practical classes with a small proportion of PBL intermixed [17, 18]. Currently, the implementation of PBL in relation to medical research in China remains in its infancy [19].

From an instructive perspective, PBL emphasises the importance of understanding not only content but also disciplinary epistemologies and investigative strategies through collaborative problem solving and sense making, reflecting on experiences, developing evidence-based explanations, communicating ideas, enhancing discipline-specific reasoning skills and engaging in self-directed inquiry [20]. To successfully use PBL, students must take responsibility for the learning process. However, for many students, this does not occur naturally or easily [21]. Previous studies have shown that PBL fosters the development of self-directed, lifelong learning *as long as students are supported and guided* [22]. Therefore, facilitation as a supporting system is central to the process of PBL [23]. This supporting system involves a rigorous, structured and flexible approach delivered by PBL tutors, whose role should be facilitative rather than didactic [24, 25]. PBL tutors are required to acquire a mixture of direct and non-directive facilitation techniques built on humanistic attitudes of education that support significant, meaningful and experiential learning [26, 27]. The directive facilitation approach is important given the complex nature of PBL; for example, a tutor may provide direct instruction on a just-in-time basis when students are experiencing difficulties [28, 29].

The non-directive facilitative approach is particularly related to the perspective of coaching psychology, which is epistemologically based on humanistic philosophy. Coaching psychology focuses on ‘enhancing well-being and performance in personal life and work domains underpinned by models of coaching grounded in established learning theories or psychological approaches’ [30, 31]. It has been studied extensively in the educational context for a variety of purposes [32] and may have pedagogical significance in education [33]. The use of coaching psychology for learning emphasises personal involvement, careful listening, acceptance, empathy and reflection to create a non-threatening and non-judgemental environment where learners feel free to delve into their own experiences and seek answers to their own problems [34].

Coaching psychologists believe that knowledge needs to be personally appropriated and that this goal can be achieved through a specific type of encounter between the coach and the learners. The responsibility for solving the problems, learning and growing rests with the learners rather than with the coach. Evidence has shown that incorporating coaching psychology into an inquiry-based learning process is beneficial in terms of optimising students’ learning experience; scaffolding the inquiry process; developing positive learning dispositions; and fostering students’ learning relationships, autonomy, self-awareness and learning agency [33].

Coaching psychology and PBL differ in various aspects in that one is a sub-discipline in the field of psychology and the other is a learning methodology. For example, coaching psychology considers the qualities and characteristics of coaches from a humanistic perspective, whereas in PBL, tutors’ personal qualities are not considered to be a main factor. However, there are several common threads running through the review of PBL and coaching psychology. Philosophically, they are both grounded in social constructionism, which is concerned with how learners construct new knowledge and build their own mental structures through social interaction with other people and their environment. Theoretically, they both explicitly stress the developmental, situated nature of the learning process and learners’ meaning making and reflection based on their self-directedness and self-determination. In practice, they both emphasise experiential learning, which refers to using an authentic method of achieving understanding through confronting problems and exploring solutions in real learning contexts. Furthermore, they both acknowledge the importance of learning facilitators, i.e., coaches, tutors or mentors, who play an essential role in supporting learners in their own learning and development. Therefore, we posit that PBL and coaching psychology share an affinity in terms of learning facilitation.

The literature on coaching in PBL is limited, although some researchers have touched on the subject. Barrows and Tamblyn offered their vision of a PBL tutor as a metacognitive coach and mentor [24]. Maudsley noted that the PBL tutor becomes both the steward of the group process and the metacognitive coach by guiding and supporting students’ learning [35] and that PBL tutors are expected to manage increasingly diverse and ambiguous roles defined as mentor, coach, model and guide [36, 37]. In addition, it is anticipated that the PBL tutor will function as a group facilitator to support students’ self-directed, active learning and foster critical thinking skills and lifelong learning habits rather than to convey knowledge [38, 39]. The tutor’s role is to coach students only when appropriate to ensure that they make optimal use of the learning opportunities and then withdraw as students develop expertise in the process while continuing to monitor the quality of learning [40].

The nature of PBL is moving towards a *learning design* and *a facilitation system*, which is the core of coaching psychology for learning [33]. We believe that a systematic visual representation of the PBL process as a collaborative endeavour between tutors and students can help medical education researchers understand the nature of PBL. This framework should be dependent on the local cultural context [41].

In the context of Chinese medical education, there are very few studies of PBL within a visualised model clarifying interrelated factors in the process. Liu and his colleagues developed a PBL model that integrates students, tutors and patients in a real clinical context [12]. Zhang constructed a PBL model applicable to basic healthcare education that emphasises the relationship between tutors and students for four modes of learning: guided learning, self-directed learning, inspired learning and motivated learning [42]. Huang and his team expanded the application of PBL and explored a ‘P^n^BL model’ involving several elements of clinical practice: Person/People, Problem, Project/Program, Product, Portfolio, Performance and Process. These are good examples of visualised PBL processes in Chinese medical education [10]. However, none of these models have the psychological underpinnings that are essential for the learning and facilitation involved in PBL. Additionally, the dynamics between the critical elements of these models are sometimes over-simplified.

This study aimed to explore a new framework that integrates essential features of current PBL and the perspective of coaching psychology in the wider context of Chinese medical education using a participatory research approach. We expected the integrated framework to be applicable to the PBL process, particularly in PBL tutorial settings. We combined research goals and action goals because they contributed to establishing a learning community and generating valid data [43]. The current study engaged all members equally as co-researchers and enabled the creation, development and modification of a new model for medical education as the result of collective wisdom.

## Methods

### Research design

The overall design was informed by participatory research. Participatory research is ‘an orientation to inquiry’ and can be regarded as a methodology that argues in favour of the significance and usefulness of involving research partners in a knowledge-production process that is reflexive, flexible and iterative [44]. Participatory research methods are not fundamentally distinct from other empirical social research approaches and are linked closely to qualitative methods [45]. The key element of participatory research is not the methods; rather, it is the attitude of researchers, which in turn determines how, by, and for whom research is conceptualised and conducted. The most important distinction between participatory research and other research methodologies is the location of power at the various stages of the research process [46]. Rooted in common principles of action research and participatory action research, participatory research emphasises listening, observing, feedback, interaction and open dialogue to establish a non-hierarchical learning community that assumes a reciprocity of influence. Through a process of mutual learning that takes place throughout the research process rather than at distinct stages, participants are included in the research as owners of their own knowledge and empowered to take action [47]. In our study, the application of participatory methodologies was collaborative and consultative in practice. We regarded participants as agents and active contributors rather than research subjects and as capable of identifying their own problems, analysing their own situation and designing their own solutions. The role of researchers was modified from directors to facilitators or catalysts. First, spaces where participants could be empowered to engage in the research process were created. Then, there was movement towards relinquishing control and developing participants’ ownership of the research question and the information that was generated, analysed, represented and acted upon in the future.

### Participants

Four educational psychology researchers and ten participants from a medical school in East China participated in the research. Participants included four second-year medical students (two females and two males) who nearly finished their first PBL module at the medical school, four final-year medical students (three females and one male) who finished a series of PBL modules during their university education and two Respiratory doctors (one female and one male) who were professionally trained as accredited PBL tutors with a minimum of three years of PBL experience. They participated in the research voluntarily and completed the entire research process. The participants were each given 500 RMB as compensation for their time and efforts.

### Research programme

The research programme consisted of four workshops held from October 2014 to January 2015. The first workshop began with an introductory session on the research framework, followed by a discussion of the current PBL curriculum in the medical school and the main concepts of coaching psychology. In the second workshop, we explored the fit between the adoption of coaching psychology and PBL, blended features of coaching into a PBL curriculum and developed draft models. The third workshop focused on investigating the advantages and the limitations of the draft models, advancing the discussion towards a more integrated model and practicing essential coaching skills and techniques. The final workshop concentrated on modifying and refining the integrated model and ended with implementation plans. During the discussions in the workshops, the research team and the participants were divided into two groups, each with a mix of students, tutors and research team members. Key learning points were summarised at the end of each workshop to aid personal reflection, provide continuity and guide participants towards the next workshop.

At the end of the research programme, participants were asked to provide feedback on the workshops. The feedback was generally very positive; participants reported a better understanding of PBL and coaching psychology, mastery of coaching skills and techniques and increased confidence in the implementation of the new model. The participants stated that they were highly engaged and motivated in the group discussion and collaborative creation of the model and felt as though they were creative and energetic. They reported that the participatory research approach provided a friendly, open and safe learning environment that allowed them to share opinions and challenged them to engage in deep thinking.

### Ethical considerations

The research protocol was approved by the Research Ethics Committee of East China Normal University and Tongji University. All participants completed informed consent forms that included an introduction of the overall programme and explanations of the commitment involved in participation. All participants were reassured of the confidentiality of the data and that their course evaluations would not be impacted if they chose to withdraw from the study. It was acknowledged that in small-scale and highly specific participatory research, it might be difficult to protect individuals’ identity. To address this concern, all members of the group signed a confidentiality agreement.

### Data collection

Data were primarily collected through qualitative methods. We video-recorded our participatory observation of the participants’ conversations, behaviours and interactional patterns in each workshop. We conducted and video-recorded four focus groups and eight semi-structured interviews and collected participants’ notes, posters, PPT and other relevant documents. These methods were complemented by the collection of some descriptive quantitative data gathered from the evaluation forms at the end of the programme. Of note, some of the data collection and analysis processes were interactive and continuous in a spiral form that enabled us to reflect on the previous analytical results and alter actions in the next phase of data collection.

### Data analysis

The data analysis process was inspired by Glaser and Strauss’ grounded theory [48] and Braun and Clark’s thematic analysis method [49] with the purpose of developing a model. The researchers independently coded interviews, documents and observational data after each workshop; performed thematic content analysis; discussed disagreement; and reached consensus on the overall analysis. We then sent our preliminary findings to the participants, invited them to offer their opinions and made the feedback sessions an integral part of the data analysis process. The model was drafted, discussed, modified and shared with the participants. In this way, we not only triangulated the sources and methods of data analysis but also deepened our understanding as researchers through the diversity of our opinions and experiences.

## Results

In this section, we briefly present the key findings that emerged from the thematic content analysis. Due to word count restrictions, direct quotes from the participants are limited and incorporated into the text.

### Current experience of the PBL curriculum

The participants had a thorough discussion about the strengths and limitations of and expected improvements to the existing PBL curriculum at the Medical School of Tongji University. The comments are summarised in Tables 1, 2 and 3 and nodes with our interpretation of the participants’ quotes are presented.

**Table 1:** Strengths of the current PBL curriculum

Theme	Sub-theme	Node
Cultivate motivation		Autonomy and agency
		Interest and intrinsic motivation
Develop abilities	Cognitive abilities	Information searching and screening ability
		Higher order thinking ability
		Foreign language ability
		Ability of mastering learning materials
		Clinical professional ability
	Comprehensive learning capacities	Self-expression and presentation ability
		Problem discovery and solving ability
		Collaboration and cooperation ability
		Flexibility and adaptation to contexts
		Independent thinking ability
		Self-correction ability
		Time management ability
		Exploration and investigation ability
		Creativity and innovation ability
		Communication ability
Advance knowledge		Expand the scope of knowledge
		Support learning of basic medical knowledge
		Update knowledge to the latest
		Foster memory of existing knowledge
		Linking new information to the prior knowledge
Build learning environment		Create positive learning atmosphere
		Encouragement

**Table 2:** Limitations of the current PBL curriculum

Theme	Sub-theme	Node
Student aspect	Individual problem	Study time is too long
		Too much pressure on study
		Unclear roles in individual student
	Group problem	Unequal participation of students
		Problem in allocation of group work
		Too many students in one group
		Lack of comparison among members
		Discussion topic slides off track
		Lack of effective collaboration
		Groups are not divided voluntarily
	Communication problem	Insufficient communication within group
		Difficulty of communication after course
Tutor aspect	Individual problem	Insufficient understanding of students
		A shortage of effective training
	Tutoring problem	Difficulty of mastering the right time to intervene
		Lack of feedback and evaluation system
		Incapable to offer effective instruction
		Incapable to provide sufficient learning resources
		Difficulty of adaptation to various instructional relationships
		Lack of control of presentation time
PBL course aspect	The nature of PBL	Unclear nature and concepts of PBL
		PBL is not suitable for all the courses
	PBL knowledge	Lack of logical system in scattered knowledge
		Rote learning is needed for inert knowledge
		Superficial learning in acquiring knowledge
		Insufficient time in learning basic knowledge
	PBL case	Case is too difficult for the students
		Case is irrelevant to the course
		Case is not interesting enough
	Assessment	Unclear assessment criteria
	Language	High requirement on English language

**Table 3:** Expected improvements of the current PBL curriculum

Theme	Node
Student aspect	Enhance team spirit and collaborative ability
	Adapt to the fast learning pace
Tutor aspect	Appropriately intervene at the right time
	Provide just-in-time instruction and guidance
	Clearly answer students’ questions
	Understand students’ psychological status
	Let students to understand tutors’ background
PBL course aspect	Improve group arrangement and structure
	An emphasis on humanistic aspects into the course
	Early adoption of PBL philosophy
	An emphasis on theory learning
	Improve PBL cases
	Improve assessment of PBL learning outcomes

### The relationships and roles of tutors and students

The tutor-student relationships were found to be interactive and multi-faceted. Generally, the nature of the relationships varied depending on *tasks* or *activities* that tutors and students undertook. The activities further defined the *roles* and *responsibilities* that both parties should take during the learning process. The main tutor-student interactions included mutual feedback, facilitation and understanding. Tutors functioned as teachers in a more traditional sense when they provided direct instruction to the students.

*My tutors were just like normal teachers when they were offering direct guidance, answering questions and summarising problems that we presented (S1).*

Meanwhile, comments on tutors’ indirect roles described them as ‘supporters’, ‘mentors’, ‘coaches’, ‘facilitators’, ‘participants’ and ‘friends’.

*We can be many different roles when we indirectly support the students, such as coaches, co-workers, friends, mentors, especially in clinical experience, and so on (T2).*

The tutors embraced the PBL philosophy and acknowledged that they were co-learners with the students.

*I like PBL a lot…I think we (tutors) are the same as students in learning. We learn together basically (T1).*

The students’ roles varied based on their individual differences, level of participation and group interaction. The students adopted roles as explorers, knowledge seekers, problem solvers, inquirers and presenters working in a team.

*We have many different roles in PBL… we need to find knowledge by ourselves rather than waiting for the tutors to tell us; we need to ask questions and come up with problems to be solved; we need to search for information and make PPT; we need to present in front of other students and the tutors. But, we all have different roles sometimes because we are all good at different things. Some of us are more talkative, more capable of finding out information… So, we do different things but we put everything together and work as a team (S4).*

### Student group dynamics

The evidence regarding student group dynamics was gathered from conversations and non-verbal behaviours in observations. The diversity (e.g., gender, educational background, personal characteristics) of individual members contributed greatly to the advancement of the participatory learning process and creativity of actions. Group members might change their perspectives on what it meant to work together towards a goal and change their understanding of their learning relationships with each other.

*I used to think that working together is like searching for information and put them together… But, we are still working on our own. Now, I feel we have the same goal and we want to produce the whole thing altogether. We work more closely and we have a kind of teamwork-based relationship that feels different than before (S2).*

When drafting the models, the students were collaborative and friendly to each other and concerned with the protection of their products. We noticed that only one student was dissatisfied with the draft model created in his group and, thus, provided an individual draft; he was encouraged by the tutors to express his thoughts to his group members and join in the collaborative work.

The tutors functioned as group facilitators to manage group dynamics and discussions. They also participated in drafting the model by sharing their experience of PBL tutoring and highlighting the critical elements that should be included in the model.

### Development of self-directed and self-regulated learning

Participants agreed that the students should develop self-directed and self-regulated learning ability, be responsible for their own learning and actively participate in the process of knowledge construction and meaning making. When students applied self-directedness and self-regulation, they developed identities as learners with ownership over what they had learned and a sense of agency in terms of how they learn.

*Learning is basically our own thing… We are taking responsibility for learning, and it is our choice to become a professional doctor in the future (S3).*

*I think the students are getting a greater sense that they are the agents in learning. Through this (research programme), they become quite active in asking questions, drawing models, and so on. They may apply that to PBL lessons too (T1).*

Some participants mentioned that their experience involved a problem of role conflict with deeply ingrained habits that they had developed through more familiar classroom experiences in which they were passive recipients of knowledge and rules in clinical practice. They reported that PBL presented challenges that allowed them to become intrinsically motivated, set goals, plan a course of action, select appropriate strategies, and self-monitor and self-evaluate their learning.

*I feel that being more self-regulated is important in the PBL course but not in other kinds of courses. We are used to sitting in big lectures since primary school and secondary school and listening to teachers. I have the habit of listening to teachers… With PBL, you can’t just listen, you need to work by yourself (S4).*

Tutors were responsible for initiating a positive learning environment that embraced, encouraged and fostered effective self-directed learning. Tutors were encouraged to implement the PBL philosophy as soon as possible, cultivate positive learning dispositions and higher-order learning skills and develop a healthy expectation of students.

*Tutors should create a good learning environment for us in the first place so that discussion can happen. Sometimes I don’t know whether I can talk because I am not sure about the tutors’ attitudes (S2).*

*I think tutors can spend some time talking about PBL, what it is, how it works, etc. We are unfamiliar with it at the beginning… And also we want to know what they really want to see in us (S1).*

### Coaching in PBL facilitation

The participants showed a great interest in coaching psychology theories, concepts, models, skills and techniques. They agreed that explicitly and intentionally adopting a coaching psychology perspective helped to clarify the tutors’ roles and processes in PBL facilitation.

*The underlying philosophy and principles of PBL and coaching psychology are basically almost the same… Being a coach is essentially similar to what PBL tutors usually are. I play different roles at different times and on different occasions (T1).*

*The knowledge of coaching psychology extends the understanding of PBL learning and facilitation (T2).*

Coaching psychology was reported to help the participants both personally and professionally.

*It (coaching psychology) helps me to communicate better during group work, pay attention to details in conversations in daily life and with patients when I become a doctor in the future (S3).*

In addition, the participants actively generated ways to employ coaching skills and techniques in various settings such as flexible usage of open and closed questions, empathetic listening, positive feedback, perspective taking, etc.

*We can use them (coaching skills) when talking with patients, listening to their problems, listening to our colleagues, answering their (the patients’) questions in a more sensitive and appropriate way, understanding their situations and imagining how we would feel if we were the patients (S2).*

Paradoxically, the students claimed that although coaching is beneficial, they still prefer some didactic learning alongside the opportunity to exercise critical thinking.

*I think tutors’ teaching is still quite important because we cannot find everything in the right way… Sometimes we waste a lot of time and do a presentation but actually miss a lot of points. When tutors talk and summarise later, we find it very helpful… Definitely the tutors need to tell us about theories and basic knowledge. But, we can challenge the tutors and question the knowledge… it shows that we are actively thinking rather than just taking in everything they say (S1).*

## Discussion

In this study, the participants discovered the meaning of coaching psychology and PBL experience, inquired about the roots of the preconceptions of PBL problems in Chinese medical education and formulated a new pedagogical framework through a series of democratic and transformative meaning-making processes. This unorthodox approach fundamentally changed the role of participants from subjects of psychological studies to co-owners of the research process and product [33, 44]. In the discussion, we illustrate the model integrating PBL and coaching psychology, the C + PBL Model, as the main product of the research programme.

The model shares some common key elements of PBL with other established models that we have reviewed. However, the model that emerged from the data has a number of innovative aspects, particularly in the Chinese medical educational context, and it appears to reflect a general, comprehensive approach rather than merely a tutorial process. There are a number of contributions that coaching psychology brings to the existing PBL approach, as indicated by the C + PBL model.

We explicitly address the following four points: i) the new model regards empathy and medical humanity as one of the key learning goals in PBL tutorials; ii) it emphasises not only the cognitive scaffolding mentioned in the earlier PBL studies but also the emotional scaffolding overlooked by previous studies; iii) it recognises the multiplicity of roles that tutors adopt during the PBL tutorial and gives more weight to the balance between the roles of knowledge experts and academic coaches; and, finally, iv) it addresses mutual feedback and communication between tutors and students in a democratic, collaborative learning environment.

These ideas may not be entirely new and have been covered in some specific PBL approaches adopted in various institutions. However, our research results and the feedback from the participants highlighted that Chinese medical tutors and administrative committees in the medical schools have not paid much attention to these points or have done so only at a superficial level. We make strong claims that these four points are essential for supporting medical students’ and tutors’ personal and professional development.

### Description of the C + PBL Model

**Fig. 1:**
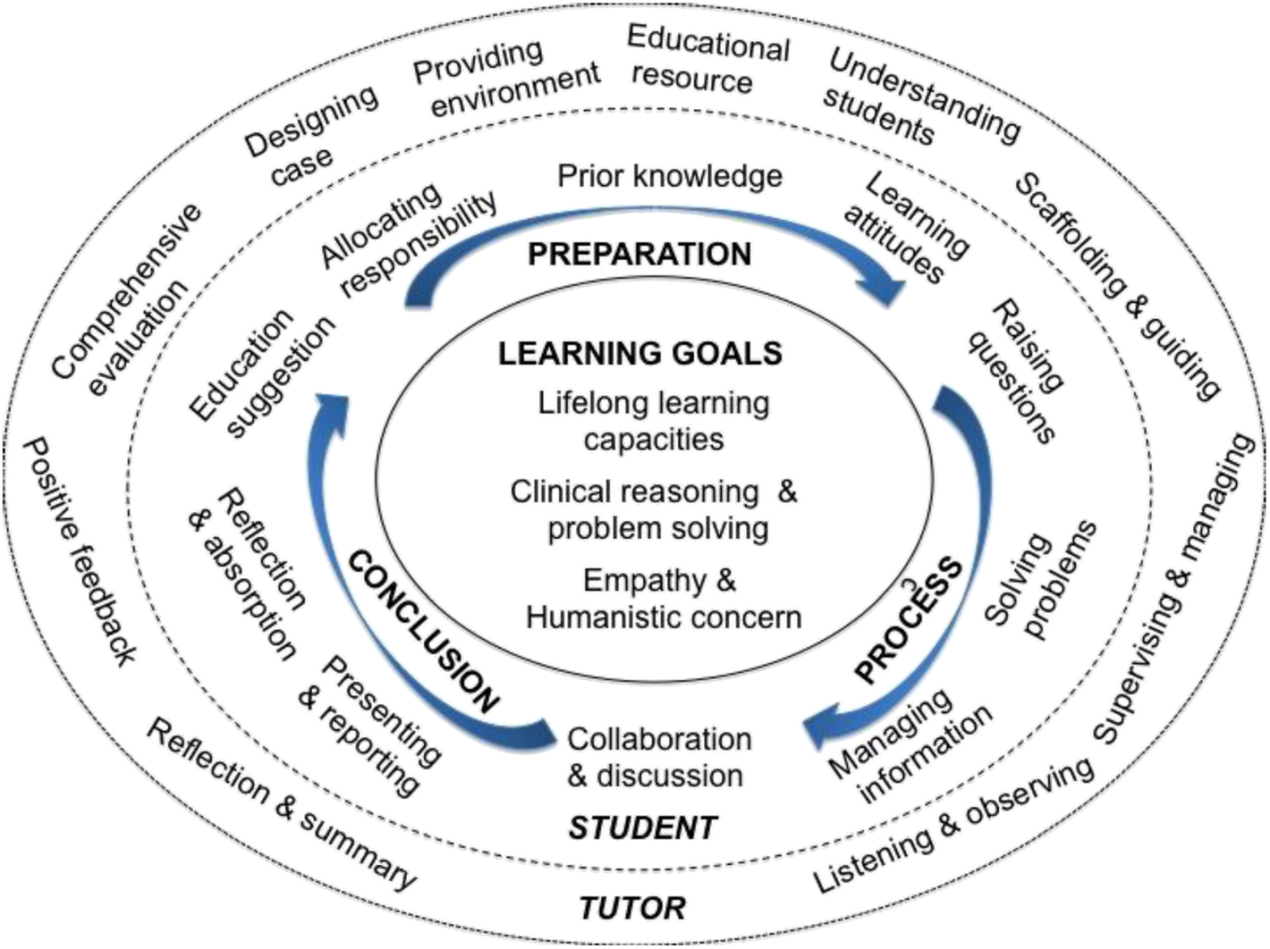
The C + PBL Model integrating PBL and coaching psychology

The C + PBL Model (Fig. 1) consists of three phases: Preparation, Process, and Conclusion, which lead to the next learning cycle. We propose that dynamic, reciprocal interconnections exist among students, tutors, PBL activities and learning goals. Given the nature of these relationships, each phase of PBL represents opportunities for both students and tutors to develop specific learning capacities. Therefore, the model describes a structure by which students and tutors can focus their efforts on achieving learning goals through a series of learning and facilitating activities.

*The Learning Goals* are as follows: 1) lifelong learning capacities, such as collaborative learning ability, communication ability, leadership, creativity, resilience, etc.; 2) reasoning and problem solving, including the abilities of understanding symptoms, identifying problems, managing knowledge and information and actively seeking solutions, which are particularly applicable to clinical settings; 3) empathy and humanistic concern, which involve demonstrating person-centred attention and warmth, responding to individual patients’ needs, being authentic and congruent in professional work and managing communication and ethical issues with the patients and their families.

*The Student and Tutor circles* are presented by broken lines that indicate that there are no barriers between students, tutors and the wider educational context. PBL activities performed by students and tutors have mutual influence. The Tutor circle seems to wrap the Student circle, representing that a tutor should facilitate and support the development of students while both groups aim to achieve learning goals. A tutor always metaphorically ‘embraces’ students, who are encouraged to take responsibility for and develop ownership of the PBL process.

*The Preparation Phase* enables students and tutors to complete necessary PBL launch tasks. It first requires the course team to redesign the PBL curriculum by radically changing the content and providing a staff development programme that introduces tutors to the messages of coaching psychology and PBL. In this phase, the tutor should initiate certain activities, including designing cases; providing a supportive learning environment; identifying and preparing necessary learning resources, including technical arrangement; and understanding students’ level of learning and, ideally, their individual background. Students’ activities include developing positive attitudes and a sense of preparedness for learning, building on prior knowledge that will serve as the basis of further intellectual development and allocating roles in learning groups.

*The Process Phase* includes students’ iterative cycle of raising and answering questions, managing information and making meaning from it, collaborating and discussing the problems. These activities should be conducted while tutors provide appropriate scaffolding and guidance, monitor the process towards the goals, manage group dynamics, listen to students’ voices and observe students’ performance. In this phase, students engage in complex learning tasks and explore their own path to solving the problems. To support students through this phase, the tutor plays the roles of coach and group facilitator*.* These two roles indicate the important incorporation of coaching psychology. The coaching role consists of scaffolding the learning process by using various learning materials, intentionally eliciting students’ articulation of thoughts and reasoning, modelling higher-order cognitive skills, using group coaching techniques to ensure students’ active participation in discussions and linking the collective activities to the learning goals.

During *the Conclusion Phase*, the students share their solutions; discuss their rationale and present their learning outcomes (typically in the form of a presentation); reflect on the new knowledge, conceptual understanding and overall learning experience; conduct peer- and self-assessment; and provide suggestions for improving the PBL curriculum in relation to the learning goals and expectations. In this phase, the tutor’s role is to conduct comprehensive assessments (both summative and formative) of students’ performance, provide positive and encouraging feedback, summarise the key learning points and reflect on what has been working well and what might be done differently in the future. It is important to note that although this phase starts with students’ learning performance and assessment, it focuses on on-going reflection and feedback, which are critical in shaping future course of actions.

### Empathy as a key learning goal

The C + PBL model explicitly considers empathy and humanistic concerns as one of the learning goals in medical education. Empathy is recognised as a central element in achieving positive health care outcomes [50, 51] and as a professional skill that a ‘good doctor’ possesses [52]. Nevertheless, the display of empathy is not consonant with Chinese medical education. According to our review of Chinese medical PBL curricula, the development of empathy is not explicitly included in any of these curricula. In this model, we adopt a three-factor model of medical empathy that consists of ‘perspective taking’, ‘compassionate care’ and ‘standing in the patient’s shoes’ [51, 53]. We add ‘skilful communication’ as the fourth factor because the three factors listed above are expressed through effective communication between doctors and patients. We believe that empathy cannot be directly *taught* in the classroom. Rather, empathy could be *coached* in a learner-centred, non-didactic way. One phenomenological inquiry on empathy suggested that one way to enhance medical students’ empathetic skills is to model these skills during medical school [54]. By displaying a willingness to listen, connect, care and engage in other empathetic behaviours towards students, the tutor could make coaching a role-modelling process that leads to students’ awareness of building rapport with patients and satisfying their psychological and affective needs in clinical practice. In addition, students should be given time and space to observe, acquire and demonstrate an empathetic disposition. However, it might be difficult to assess empathy because structured clinical examinations typically do not provide a wealth of opportunities to develop empathy [53]. We will return to the issue of evaluation later in this paper.

### Authentic learning experience

An *authentic learning experience* is highly valued and promoted in this framework. Authenticity is connected with its dynamic and educative character as well as its capacity to promote inquiry skills, provide a sense of freedom and form a self-directive purpose [55, 56]. In addition, the authentic problem-solving experience is embedded in meaningful contexts because real-world practice is more suffused with complex and ill-structured problems [57, 58]. The authentic experience should be defined and owned by the students. The tutor could enhance this experience by preparing an appropriate problem scenario, allowing students’ to have a voice and choice in conducting the inquiry strategically, understanding each student’s learning needs, facilitating dialogues among small groups, stimulating students to integrate new learning content with previous knowledge and encouraging students to step out of their comfort zone [21].

### Three modes in learning-centred coaching

The C + PBL model indicates a learning-centred coaching process that is similar to what Gyori called a ‘three-mode process of mentorship’ [59]. It consists of bottom-up (modelling and scaffolding), lateral (collaborating and engaging) and top-down (organising and supervising) processes for sharing autonomy and participating in deep learning. Regarding the bottom-up process, the tutor needs to demonstrate how to perform clinical problem solving through behaviour modelling [60–62] and scaffold the learning tasks to foster students’ metacognitive capabilities. The lateral process allows co-learning between the tutor and the students to emerge organically in a highly collaborative environment. The top-down process concerns promoting students’ self-directed learning to generate more questions, reach for the answers and discover the answers through a process of systematic inquiry as well as maintaining important professional standards. These three processes are interrelated in the model; for instance, when working with students, the tutor models effective collaboration and simultaneously supervises the process of collaboration in which they are participating.

### The tutor as a knowledge expert and group facilitator

This model emphasises that the tutor needs to act as a coach, playing a balanced role as an expert in both the subject matter and in facilitating students’ learning process [59, 60]. The tutor should demonstrate social congruence [63–65] to communicate informally and empathetically with students, create a learning environment that encourages the open exchange of thoughts and, thus, directly affect group functioning and student achievement. It is found that tutors with subject content expertise are more inclined to play a directive role in the tutorial process [66]. Although content experts have positive effects on student learning, it may be beneficial for these experts to develop knowledge of when and how to use this expertise to facilitate learning. Most Chinese PBL tutors are doctors or clinicians in a specific area. They are content experts in certain fields but have very limited facilitation and coaching skills training. Therefore, the development of a broad range of strategies, including coaching, to stimulate student learning and encourage optimal group functioning should be a major focus of tutor recruitment and training.

### Cognitive and emotional scaffolding

To successfully apply the C + PBL Model, the tutor should acquire the basic attitudes, beliefs, characteristics and skills necessary for any PBL curriculum, such as supporting metacognitive development and strategic and reflective questioning; modulating the level of challenge of the learning to meet student requirements; and monitoring students’ educational progress and group dynamics [67–69]. In addition, *cognitive and emotional scaffolding* is at the heart of coaching psychology for learning [33]. Scaffolding refers to the temporary support provided to learners (by another person who might be more capable) to complete a task that they would not be able to complete independently and to facilitate the learners’ zone of proximal development [70]. Scaffolding should be gradually withdrawn as the students become increasingly competent and responsible for their own learning [71–73].

From a cognitive perspective, students need to integrate, possess and apply a large amount of domain knowledge during the problem-solving process; thus, students’ cognitive loads are expected to increase over the course of PBL [58]. Cognitive scaffolding can reduce cognitive loads by transforming difficult and complex problems into more manageable and accessible tasks, providing predictable ways to move through activity structures, restricting the options available to the learners and setting social norms for participation and the use of resources [74, 75]. Moreover, cognitive scaffolding helps students acquire disciplinary ways of thinking and acting, supports mindful and productive engagement with the learning process, models questions that students need to be asking themselves, provides a framework for students to construct knowledge independently, offers explanations when needed and problematizes important aspects of students’ work to force them to engage with key disciplinary frameworks and strategies [76–80]. It is associated with coaching dialogues for assessing the level of students’ thinking and moving it through a systematic series of questions [81].

Emotional scaffolding is important for guiding students through the frustration, lack of confidence, emotional issues and teamwork problems that they might experience during PBL [82]. There is a focus on the nondirective facilitation of emotional scaffolding regarding self-determination and actualisation tendency [83]. The students involved in PBL are considered the experts on their own affective needs, and the tutor is considered an expert only on maintaining the attitudinal conditions and managing rapport in the relationship with the students rather than an expert on the students or how the students should learn [84–87]. Emotional scaffolding includes being a friend, a colleague, a mentor, a coach, a role model or a counsellor to the students [88]. The tutor’s responsibility is to use active and empathetic listening, understand students’ available educational and social backgrounds, respect and value students’ viewpoints with a non-judgemental attitude, demonstrate outstanding communication skills and hold beliefs consistent with the humanistic approach.

Due to the complex and multi-skilled nature of scaffolding, some tutors are unsure of how to determine the appropriate time to intervene and how the balance between too much and too little structure with each unique PBL group. Successful scaffolding is largely dependent on the availability and skills of tutors who know when and how to adopt facilitation strategies [89, 90]. The desirable attributes, skills and strategies commonly used in personal and professional coaching include providing feedback, using open or closed questions, eliciting students’ explanations, elaborating students’ thinking, and supporting students’ reflection and expression of their thoughts and feelings [91, 92].

### Mutual feedback between the tutor and the students

The C + PBL Model emphasises mutual feedback between the tutor and the students. The on-going feedback can be categorised into two types: specific feedback and general feedback. Specific feedback refers to asking questions to prompt thinking instead of providing direct guidance or correcting errors, offering suggestions and pointing the students to a specific resource for additional information about certain concepts. This kind of feedback requires the tutor to observe students’ level of understanding and respond accordingly in a student-centred environment [93, 94]. General feedback needs to be non-threatening and mastery-oriented to enhance self-directed learning [95], a sense of agency and ownership over learning [96]. This type of feedback is provided to empower students with intellectual responsibility [97] and improve their self-efficacy beliefs [98]. The students could evaluate the tutor’s performance but such evaluation should be carried out with caution because less experienced tutors may engage in behaviours aimed at pleasing students. Because the tutor’s and students’ expectations might differ, the tutor’s roles and duties must be made explicit to both parties from the outset [88]. Feedback cannot be given without mutual trust between the tutor and students; therefore, it is important to establish rapport and connection at the beginning of the process and to maintain this trust throughout the process [87].

### The challenges of the new framework

The main challenges of the new framework involve implementation and evaluation issues. Although the participants felt confident in the successful implementation of the model, incorporating it into the existing medical educational system is a great challenge that correlates, to some extent, with reported difficulties (e.g., too time-consuming, lack of staff, students might feel compulsion) in implementing PBL in Asian countries [99, 100]. Additional challenges in the implementation include building a solid knowledge base of coaching psychology, PBL and clinical practice; creating a nurturing and supporting culture using coaching principles; and enhancing students’ self-directed learning and tutors’ continuous professional development using coaching strategies. Implementing the model could be a daunting task requiring strong support from academic administrators, well-trained and committed tutors, skilful and dedicated case writers, appropriate technical support and well-prepared students with a belief in the PBL philosophy.

The alignment between learning activities and assessments is crucial for participation in PBL [101]. Whether the fusion of coaching psychology and PBL is effective is a big question, and we need to find evidence from various aspects to view a whole picture. The learning dispositions, reasoning and problem-solving skills, and empathy that we expect students to develop are long-term learning goals and very difficult to measure. Therefore, obtaining academic or attainment results in the traditional sense is not applicable, and simple quantitative measurements could be flawed. We propose a mixed-methods approach to evaluation that comprises both quantitative and qualitative data (classroom observations, interviews, peer-assessment, narratives and learning portfolios) to give a comprehensive picture of the effectiveness of the new model.

## Conclusions

This article describes the process of developing a new framework integrating features of PBL and the perspective of coaching psychology through a participatory research approach within the context of Chinese medical education. It attempts to develop a systematic model that leads to more efficient, satisfying and long-lasting professional capacities that increase throughout the effective PBL tutorial process. We have successfully fulfilled the aim by presenting the following strengths. The participatory and dialogic research approach offered the participants opportunities to be creative, collaborate and take personal responsibility and, in many ways, resembled some of the features of their professional clinic-related practice. The triangulation of data collected from observations, focus groups, interviews and documents as well as the independent coding during data analysis enhanced the credibility and trustworthiness of the research. In this paper, we provide a rich description of an integrated model of PBL and coaching psychology. The C + PBL Model differs from the current PBL approach in that it explicitly addresses empathy and medical humanity as one of key learning goals in medical education, the tutors’ roles as knowledge experts and learning coaches for the students, cognitive and emotional scaffolding strongly grounded in psychological theories and effective mutual feedback between the tutors and students. Although it is in its infancy, the new model could be regarded as giving a sense of achievement and direction for further implementation. We anticipate that our investigations are useful in two ways. First, the C + PBL Model could serve to stimulate consideration and debate as institutions develop their own PBL concepts and procedures. Second, our study provides insights into incorporating coaching skills into professional development programmes for PBL tutors and PBL curricula for students.

In addition to its strengths and contributions, our study is subject to several limitations. A comparatively small group of medical students and tutors participated in the research programme; thus, the findings might not be generalisable to other research fields. The participatory research workshops generated a large amount and diversity of data that might be overwhelming to analyse. The third limitation was the restricted resources and time and participants’ different agendas, which made it difficult for us to include all the participants in the final data analysis stage. However, the research could be seen as one part of a collective, on-going learning journey and we would use the feedback from the participants to improve the research programme in the future.

An important next step of this study is to closely investigate the process of how tutors and students incorporate the framework into their learning using real clinical cases, ideally comparing a group that adopts the C + PBL Model with a group that follows a traditional PBL curriculum. A fine-grained examination of the process of each phase of the model, with video analysis documenting tutors’ interventions and students’ responses, thematic analysis of contextually designed questions and statistical analysis of measurements on the development of learning dispositions and capabilities, is needed. This topic deserves substantial attention and continuous efforts to advance the PBL approach in Chinese medical education.
